# Identification of an RNA-Binding-Protein-Based Prognostic Model for Ewing Sarcoma

**DOI:** 10.3390/cancers13153736

**Published:** 2021-07-25

**Authors:** Yi Chen, Huafang Su, Yanhong Su, Yifan Zhang, Yingbo Lin, Felix Haglund

**Affiliations:** 1 Department of Oncology-Pathology, Karolinska Institutet, Solna, 17176 Stockholm, Sweden; yanhong.su@ki.se (Y.S.); yifan.zhang@ki.se (Y.Z.); yingbo.lin@ki.se (Y.L.); felix.haglund@ki.se (F.H.); 2 Clinical Pathology and Cancer Diagnostics, Karolinska University Hospital, Solna, 17176 Stockholm, Sweden; 3 Department of Radiation and Medical Oncology, The First Affiliated Hospital of Wenzhou Medical University, Wenzhou 325000, China; suhuafang@wzhospital.cn

**Keywords:** Ewing sarcoma, RNA-binding proteins, regulation network, prognosis prediction, risk model

## Abstract

**Simple Summary:**

Ewing sarcoma (ES) is an aggressive childhood tumor for which response to chemotherapy is central to long-term prognosis, but few prognostic markers have been identified. RNA-binding proteins (RBPs) are strong regulators of cell behavior, working, for example, through post-translational modifications of mRNA. In this study, we investigated whether patterns in the RBP levels were related to outcomes in ES patients. A total of three distinct patterns were recognized, and additional modelling suggested that 10 RPBs had predictive value, suggesting that this model could be used in a clinical setting to identify patients with a higher risk of mortality.

**Abstract:**

RNA-binding proteins (RBPs) are important transcriptomic regulators and may be important in tumorigenesis. Here, we sought to investigate the clinical impact of RBPs for patients with Ewing sarcoma (ES). ES transcriptome signatures were characterized from four previously published cohorts and grouped into new training and validation cohorts. A total of three distinct subtypes were identified and compared for differences in patient prognosis and RBP signatures. Next, univariate Cox and Lasso regression models were used to identify hub prognosis-related RBPs and construct a prognostic risk model, and prediction capacity was assessed through time-dependent receiver operating characteristics (ROCs), Kaplan–Meier curves, and nomograms. Across the three RBP subtypes, 29 significant prognostic-associated RBP genes were identified, of which 10 were used to build and validate an RBP-associated prognostic risk model (RPRM) that had a stable predictive value and could be considered valuable for clinical risk-stratification of ES. A comparison with immunohistochemistry validation showed a significant association between overall survival and NSUN7 immunoreactivity, which was an independent favorable prognostic marker. The association of RBP signatures with ES clinical prognosis provides a strong rationale for further investigation into RBPs molecular mechanisms.

## 1. Introduction

Ewing sarcoma (ES) represents the second-most common primary bone malignancy affecting children and adolescents, with an incidence of 2.9 per million/year [1,2,3]. It is an aggressive tumor typically characterized by a fusion of the Ewing sarcoma breakpoint region 1 (EWSR1) with an erythroblast transformation specific (ETS) transcription factor gene, most frequently (>95%) the friend leukemia virus integration 1 (FLI1) gene [4,5]. Most types of ES harbors the t (11;22) (q24;q12) chromosomal translocation leading to the EWS–FLI1 fusion transcript. Genome-wide association studies identifying molecular features and genetic profiles suggest a generally low mutational burden for ES [6,7,8]. The STAG2 gene is the most frequently mutated (17%) compared to normal paired cases, followed by CDKN2A (12%), TP53 (7%), and EZH2 (2.7%). Genetic lesions of STAG2 and TP53 and deletions of CDKN2A are mutually exclusive and associated with the worst ES prognosis [6,9,10]. Recently, the Euro-Ewing 99 clinical trial revealed 3-year and 8-year overall survival (OS) rates of 72–78% and 56–65%, respectively [11]. Patients with relapsed and advanced ES have a dismal prognosis, especially when the disease has metastasized, and a short survival time is expected [12]. Therefore, prognostic biomarkers and novel intervention targets are urgently needed.

RNA-binding proteins (RBPs) are a class of proteins that interact with a series of RNAs: messenger (mRNAs), ribosomal (rRNAs), non-coding (ncRNAs), micro (miRNAs), transfer (tRNAs), small nuclear (snRNAs), and small nucleolar (snoRNAs). They exert their functions by associating with their RNA targets and generating ribonucleoprotein complexes that regulate transcription, RNA maturation, translation, and metabolism and maintain post-transcriptional genome integrity [13,14,15,16,17]. Currently, more than 1500 RBPs have been identified by large-scale quantitative methods accounting for about 7.5% of all protein-coding genes in the human genome [18,19]. EWS is a multifaceted RBP that has been implicated in modulating transcription, pre-mRNA splicing, and, importantly, in causing epigenetic remodeling in the tumorigenesis of ES [20,21]. Another example is IGF2BP3, a novel post-transcriptional regulator responsible for poor survival probability [22,23]. Our group previously reported that a higher expression of RBPs related to rRNA metabolism and mRNA splicing were significantly overrepresented in ES patients who had first-line treatment failure [22]. Thus, we hypothesized that a systematic study to explore prognosis-related RBPs would increase our knowledge of treatment resistance and facilitate risk-stratification.

In this study, we obtained ES gene expression data from previously published GEO datasets and investigated the RBP landscape in ES associated with patient outcomes. Next, the prognostic value of a 10-RBP expression signature was validated in an independent cohort. We also identified *NSUN7* as a novel independent prognostic factor that could be a diagnostic and therapeutic target.

## 2. Material and Methods

### 2.1. ES Data Processing

Both the ES gene expression microarray data and clinical data were derived from the NCBI Gene Expression Omnibus (GEO) database: GSE63155 (46 ES cases, follow-up time: median 1876 days, range 155–3987 days) [24], GSE63166 (39 ES cases, follow-up time: median 2085 days, range 286–4500 days) [24], GSE17679 (32 ES cases, follow-up time: median 1159 days, range 138–5766 days) [25], and GSE34620 (38 ES cases, follow-up time: median 1549 days, range 270–4017 days) [26]. The patient characteristics involved in this study are shown in Table 1. In these four datasets, Affymetrix HuEx1.0 (GPL5175) and Affymetrix HG-U133 Plus 2.0 (GPL570) platform data were merged into one integrated dataset. Batch effects were removed using the ComBat function of the R package surrogate variable analysis (sva) [27], and expression values were quantile-normalized across different samples. A total of 85patients from GSE63155 and GSE63166 were involved in the training cohort, while 70 from GSE17679 and GSE34620 were included in the validation cohort. We obtained a comprehensive RBP catalog consisting of 1542 genes and 318 transcriptional factors (TFs) for further analysis [19,28]. Unsupervised clustering was performed using the R package ConsesusClusterPlus to classify patients into distinct subtypes according to RBP expression. 

### 2.2. Identification of Differentially Expressed RBPs

The R package linear models for microarray data (LIMMA) [29] was used to perform differentially expressed RBP (DERBP) analysis in the training cohort by comparing clusters of patient subtypes that were significantly associated with overall survival. A false discovery rate (FDR) of <0.05 or *p*-value of <0.05 was set as the cut-off criterion. A Venn diagram was used to determine the overlapping DERBPs between two sets of comparisons. Additionally, the DETFs and DEHallmarks were generated based on the same criteria.

### 2.3. Protein–Protein Interaction (PPI) Network Construction and Functional Enrichment Analyses

To further explore the potential molecular functions, the DERBPs were submitted into the Search Tool for the Retrieval of Interacting Genes (STRING) database for building the protein–protein interaction (PPI) network [30]. The PPIs with combined scores ≥ 0.7 were selected, after which the network was constructed and visualized using Cytoscape software (version 3.8.2) (National Institute of General Medical Sciences, Bethesda, MD, USA) [31]. Any genes with a connectivity of ≥1 (node/edge) were screened as hub genes for downstream analyses. The top 50 central nodes were ranked by The MCC (maximum clique centrality) algorithm of the CytoHubba plug-in [32] in Cytoscape. The functional enrichment analyses were performed using the ClueGO and CluePedia plug-ins in Cytoscape [33,34]. The main parameters of the constructing network with ClueGO were as follows: marker list, homo sapiens; ontologies/pathways, GO (biological process) and REACTOME pathways; GO tree interval, level = 6; GO term/pathway selection, min # genes = 6 and % genes = 6.000; and GO term/pathway network connectivity (kappa score) = 0.45. Only GO terms/pathways with *p* < 0.01 were selected.

### 2.4. Prognosis-Related RBPs and the Interaction with TFs

After combining the expression levels of the hub genes with the survival status and follow-up time, a univariate Cox proportional regression analysis was used to determine the hub genes related to overall survival in the training cohort. The association between the expression level of significant survival-related genes and DETFs was assessed by Pearson correlation analysis. A correlation coefficient of ≥0.4 and *p*-value of ≤0.05 were set as the cut-off criteria. The Cytoscape ClusterViz plug-in [35] was employed to identify biological network modules between RBPs and TFs. 

### 2.5. Establishment and Validation of an RBP-Associated Prognostic Risk Model (RPRM)

The survival-related genes were subjected to penalized multivariate Cox proportional hazard survival modeling by an algorithm for variable selection based on L1-penalized Least Absolute Shrinkage and Selection Operator (LASSO) regression [36]. The process of prognostic model construction was repeated for the combination of parameter values for 1000 iterations. Subsequently, the resulting models were combined through cross-validation. The risk score formula for each sample was calculated as follows:

Risk score = β1 ∗ Exp1 + β2 ∗ Exp2 + βi ∗ Expi

where β represents the coefficient value, and Exp represents the gene expression level. Subsequently, A time-dependent receiver operating characteristic (ROC) curve was performed to evaluate the model performance by calculating the area under the curve (AUC). As a result, patients were classified into high- or low-risk groups according to the median risk score. The RBP-associated prognostic risk model (RPRM) was externally validated by the validation cohort (GSE17679 and GSE34620) to access its generalizability. Afterwards, we developed a nomogram-combing gene expression to improve risk stratification and quantify the risk assessment for survival probability at one year, three years, and five years in individual patients.

### 2.6. Assessment of Gene Expression Level and Prognostic Significance in RPRM

Our group had previously identified several pathways that were significantly associated with first-line treatment failure in ES [22]. Here, a single sample gene set enrichment analysis (ssGSEA) was used to define the estimated enrichment score of the gene signature for each sample using gene set variation analysis (GSVA) in R package [37]. Hallmark gene sets were downloaded from the Molecular Signatures Database v7.4. Gene expression levels in RPRM were assessed between dead and live patients. Significant genes were cross-validated by the validation cohort and our ES RNA-seq data.

### 2.7. Immunohistochemistry

We investigated the expression of NSUN7 in ES tumor tissue from patients (*n* = 24) who underwent standard treatment protocols (ISG/SSG III or EuroEwing99/2012 protocols) [38]. These were therapy-naive preoperative biopsies (*n* = 9), but if these were unavailable for analysis, viable tumor cells from a resection specimen (*n* = 11) or metastatic specimen (*n* = 4) were used.

Immunohistochemical staining for *NSUN7* was done using a manual protocol with a polyclonal antibody (anti-*NSUN7*, Product #PA5-54257 from ThermoFisher Scientific, Waltham, MA, USA) at 1:50 dilution. Formalin-fixed paraffin embedded (FFPE) tissue was sectioned in 4 um thick sections and deparaffinized. Antigen retrieval was performed for 10 min in low pH (citrate). Slides were incubated with the primary antibody at 4 °C overnight, followed by incubation with a biotinylated secondary antibody and signal generation using the VECTASTAIN^®^ Elite ABC-HRP Kit (Vector Laboratories, Burlingame, CA, USA).

Two clinical pathologists evaluated the staining, and after agreeing on a scoring method, the slides were scored independently in a blinded fashion. The agreement was substantial (82.6%, Cohen’s K 0.620). There was disagreement in four cases, but consensus was reached after a second review. The slides fit into three scoring categories:

- Negative (“Most of the tumor cells were completely negative”): >75% completely negative, <25% with very weak cytoplasmic staining);
- Weak (“Cases exhibited a diffuse weak staining with small areas with negative or stronger immunoreactivity”): >75% of tumor cells exhibited weak immunoreactivity); and
- Moderate (“Cases showed stronger cytoplasmic immunoreactivity in most of the tumor cells, but smaller areas with either weaker or negative immunoreactivity were sometimes observed”): >75% of the tumor cells showed moderate cytoplasmic reactivity.

Since the number of patients was limited, the staining groups were compared in two different fashions: all groups (negative vs. weak vs. moderate) or simplified (negative vs. positive).

### 2.8. Statistical Analysis

Normally distributed variables were analyzed with an unpaired two-tailed Student’s *t*-test, whereas a Mann–Whitney U test or a Wilcoxon rank-sum test was used for non-normally distributed variables. The relationship between the parameters was evaluated using the Spearman rank correlation coefficient. A log-rank test Kaplan–Meier curve and a Cox proportional hazard regression were performed for survival analysis using the R package “survminer” and “survival”. Additionally, L1-penalized LASSO regression and ROC curves were conducted using the R package “glmnet” and “pROC”, respectively [39,40,41]. R version 4.0.3 (R Foundation for Statistical Computing, Vienna, Austria) was used to execute all statistical tests and plots.

## 3. Results

### 3.1. Data Preprocessing of the ES Dataset

Data from 155 ES patients with complete follow-up and expression data from GSE61355, GSE63156, GSE34620, and GSE17679 were bioinformatically pretreated to remove batch effects. The 15,016 overlapping genes across the four GEO datasets are depicted in the Venn diagrams in Figure S1A. Data before and after normalization were carefully inspected by principal component analysis (PCA), suggesting the batch effect was successfully removed using ComBat (Figure S1B,C). In addition, the gene expression profiles were visualized in box plots to show the impact on batch effect removal (Figure S1D,E).

### 3.2. Identification of DERBPs in ES Patients and Transcriptional Subtypes

**Figure 1:**
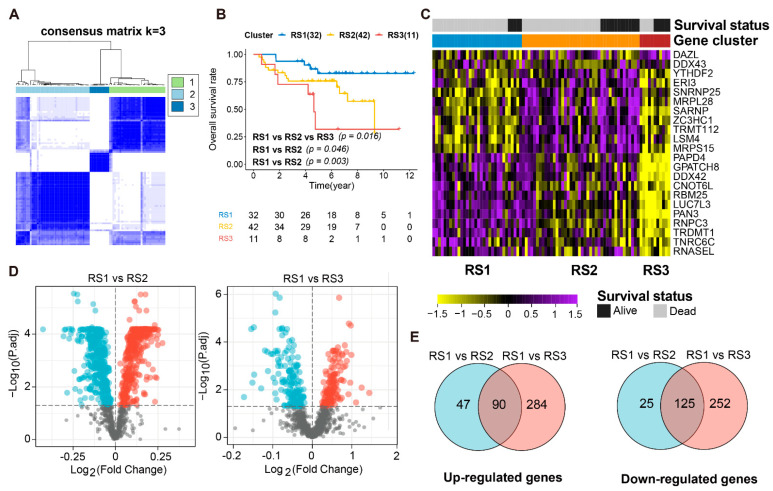
DERBP identification and the transcriptional subtypes of ES (**A**). Consensus matrices of identified clusters (k = 3). (**B**). Kaplan-Meier curves show the overall survival in ES patients among the three subtypes (*p* = 0.016, *p* = 0.045, *p* = 0.003 for RS1 vs. RS2 vs. RS3, RS1 vs. RS2 and RS1 vs. RS3, respectively). (**C**). Abundances of 22 DERBPs (identified in dead ES patients) in the three RSs. (**D**). Expression profile of RBPs between RS1 versus RS2 and RS1 versus RS3, respectively. Red and blue dots represent upregulated and downregulated RBPs in RS2 or RS3, respectively (FDR < 0.05). (**E**). Overlap up-regulated and down-regulated DEREPs between RS1 versus RS2 and RS1 versus RS3.

To investigate how RBPs affect the prognostic significance in ES, we compared the gene expression between dead and alive patients using the Wilcoxon rank-sum test (*p* < 0.05) in the training cohort. This analysis identified 22 DERBPs, and the consensus k-mean clustering on the 22 RBP signatures clearly divided ES patients into three main subtypes with clustering stability decreasing for k = 2 to 6 (Figure 1A and Figure S2). They were clustered into RS1 (*n* = 32), RS2 (*n* = 42), and RS3 (*n* = 11) with distinct prognostic differences. Interestingly, patients in Cluster 1 had the best clinical prognosis, whereas patients in Cluster 3 had the poorest (log-ranktest, *p* < 0.05) (Figure 1B). Following this, a heatmap visualized the expression levels of 22 RBPs in three RBP subtypes with survival status, where most were RS3s dominated by down-regulated transcripts (Figure 1C). Among 1542 RBPs quantified in 85 ES patients with two comparisons (RS1 vs. RS2, RS1 vs. RS3), 377 (24.4%) were significantly down-regulated, and 374 (24.3%) were significantly up-regulated in RS3 compared to RS1; 150 (9.7%) were significantly down-regulated, and 137 (8.9%) were significantly up-regulated in RS2 compared to RS1 (Wilcoxon rank-sum test, FDR < 0.05; Figure 1D). A total of 90 overlapping up-regulated and 125 overlapping down-regulated DERBPs were identified by the two comparisons and selected for further analysis (Figure 1E).

### 3.3. Functional Enrichment Analysis and PPI Network of DERBPs

**Figure 2:**
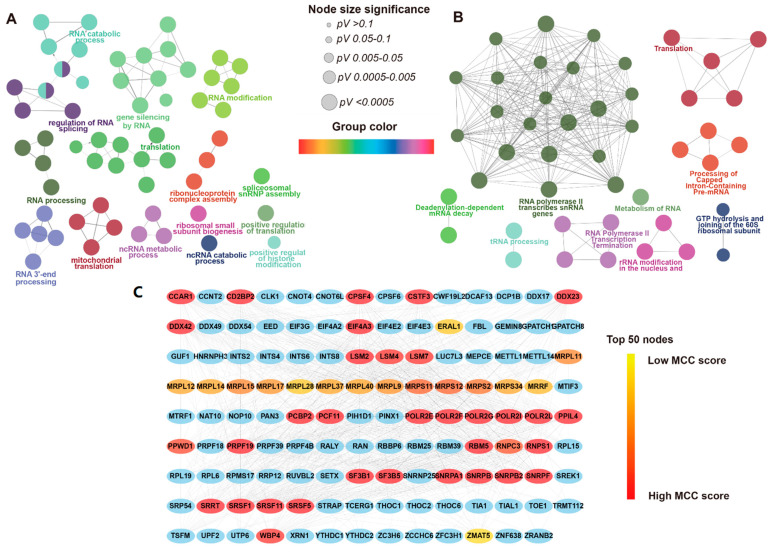
Functional enrichment analysis of DERBPs and PPI Network (**A**). GO terms (biological process) grouped network (kappa score levels ≥ 0.45, *p* < 0.01). Ellipse represents the GO terms. The node size represents the significance of the term enrichment (*p*-value), and the colors represent different functional groups. (**B**). The REACTOME pathway grouped network (kappa score levels ≥ 0.45, *p* < 0.01). Ellipse represents the GO terms. The node size represents the significance of the term enrichment (*p*-value), and the colors represent different functional groups. (**C**). The MCC score of top 50 genes in the PPI network of DERBPs (combined score ≥ 0.7) is represented by a red to yellow gradient.

Among the DERBPs, we wanted to identify the key components using co-variation analysis. Functional enrichment analysis was performed for the 218 overlapping DERBPs: 90 up-regulated, 125 down-regulated, and 3 with distinct expression profiles. Gene ontology (GO) biological process and REACTOME pathway analyses were applied to clarify the DERBP gene function. As shown in Table S1, most of the DERBPs were enriched in the GO categories of RNA metabolism (i.e., RNA processing, RNA metabolic process, and RNA catabolic process) and protein metabolism (i.e., translation, ribonucleoprotein complex assembly). In addition, enrichment analysis of the REACTOME pathways revealed that RNA metabolism and mRNA splicing and translation were mostly enriched for DERBPs (Table S2). The GO term/pathway network is shown in Figure 2A,B (*p* < 0.01). Next, we built a high-confidence PPI network that contained 171 nodes (genes) and 1234 edges, which were selected for further analysis. To identify the top 50 hub nodes, the cytoHubba plug-in was applied, and key genes were selected from the PPI network according to the MCC score (Figure 2C). Detailed gene descriptions and connectivity degrees of the top 15 hub genes are shown in Table S3. EIF4A3 was identified as having the largest number of edges interacting with 54 other genes, followed by POLR2F and SRSF1.

### 3.4. Prognosis-Related RPBs and the Regulatory Network

**Figure 3:**
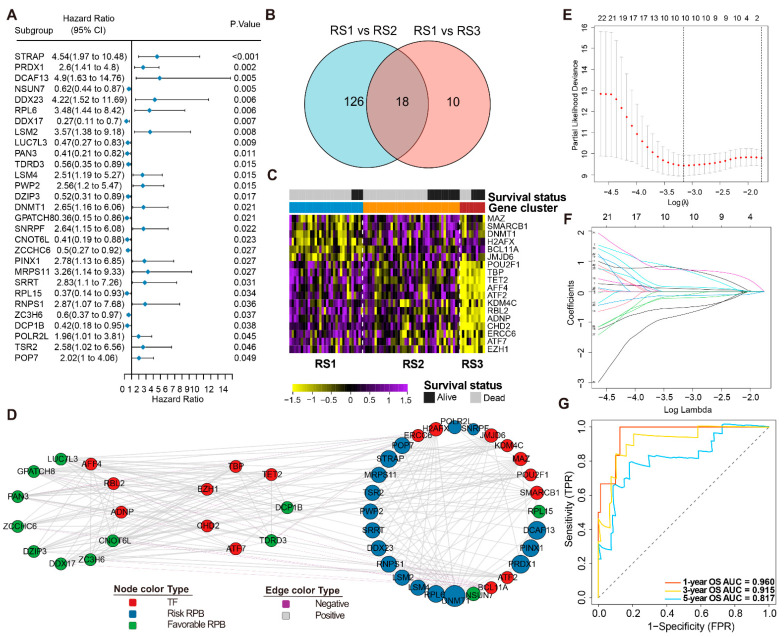
RBP regulatory network and prognostic risk model (**A**). Univariate analyses of 29 significant DERBPs with overall survival (*p* < 0.05). (**B**) Abundances of 18 DETFs (identified in dead ES patients) in the three RSs. (**C**) Overlap of DETFs between RS1 versus RS2 and RS1 versus RS3. (**D**). DERBPs network in ES. Circles represents all genes. Red indicates TF; blue indicates high risk RBP; and green indicates low risk RBP. The size of each circle indicates the degree of correlation. Grey lines indicate positive correlations, and purple lines indicate negative correlations (r > 0.4, *p* < 0.01). (**E**). Partial likelihood deviance under each log (lambda) was drawn in a LASSO Cox regression model. (**F**). The change trajectory of each independent variable in the model. (**G**). ROC curve of the prognostic values of the RPRM risk model in training group in 1-, 3- and 5-year OS.

To investigate the prognostic significance of these 171 RBPs involved in the PPI network, a univariate Cox regression analysis was performed from which 29 prognostic-associated hub RBP genes were obtained: 12 having favorable factors with a hazard ratio (HR) of <1, and 17 having risk factors (HR > 1) (Figure 3A).

Next, we identified DETFs across the three RSs to explore the regulatory mechanisms of these RPBs. We found 144 TFs significantly expressed in RS3 compared to RS1, and 28 TFs significantly expressed in RS2 compared to RS1 (FDR < 0.05). The overlapping 18 DETFs in the two comparisons were then selected (Figure 3B). A heatmap was constructed to indicate their expression levels in three subtypes with survival status (Figure 3C). We consequently created a regulatory network based on these 18 TFs and our 29 prognosis-related RPBs. A correlation score of more than 0.4 and *p* < 0.01 were set as the cut-off values. The TF-based regulatory network topology was grouped into three clusters through the ClusterViz plug-in in Cytoscape. Notably, the schematic clearly illustrated the regulatory relationships among these TFs and RBPs (Figure 3D, Table S4). 

### 3.5. Construction and Validation of the RPBs-Associated Prognostic Risk Model (RPRM)

**Figure 4:**
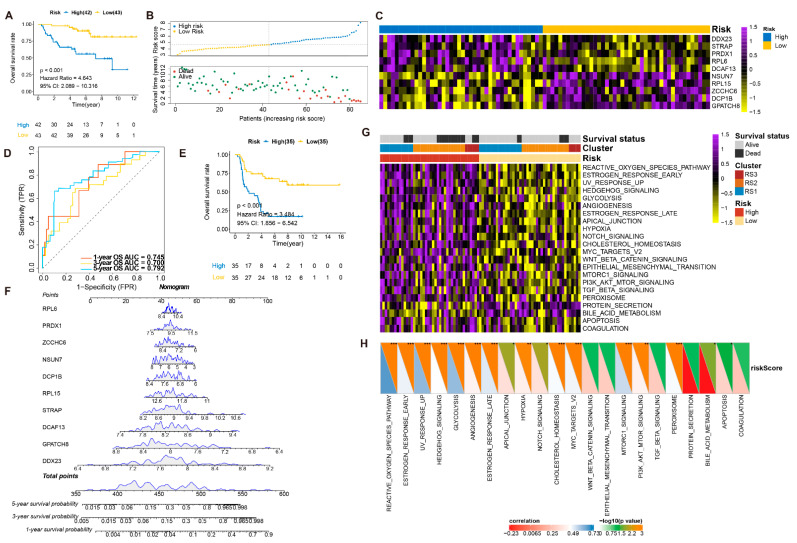
Validation and assessment of RPRM (**A**). Kaplan–Meier curves show overall survival between high-risk and low-risk patients in the training group (*p* < 0.001). (**B**). Distribution of risk score and survival time of patients in the training group. The patients were divided into high-risk and low-risk subgroups based on the median value of the risk score. Blue and yellow dots represent high- and low-risk patients, respectively. In the plot below, red and green dots indicate dead and live patients, respectively. (**C**). Abundances of 10 significant RBPs (involved in RPRM) in the training group. (**D**). ROC curve of the prognostic values of RPRM risk model in 1-, 3- and 5-year validation groups. (**E**). Kaplan–Meier curves show overall survival in validation groups between high- and low-risk patients (*p* < 0.001). (**F**). Nomogram for predicting 1-, 3-, and 5-year survival probability of ES patients in the training group. The total score of these genes for each patient is on the total points axis, which corresponds to the survival probabilities plotted on the three axes below. (**G**). Abundances of 23 DEHallmarks between high- and low-risk patients in the three RSs. (**H**). The correlation between LDA score and enrichment scores of 23 DEHallmarks. * *p* < 0.05; ** *p* < 0.01, *** *p* < 0.001.

After identifying the 29 prognosis-related RBPs, 1000 iterations of LASSO-penalized multivariate modeling were constructed, which led to 10 features with a non-zero, coefficient-based-risk model called RPRM (Figure 3E,F). We then computed the RPRM for each patient based on the risk score formula. The ROC curve exhibited significant prognostic performances for the AUC (1-year, 3-year, and 5-year OS predictions were 0.960, 0.915, and 0.817 in the training cohort and 0.745, 0.700, and 0.792 in the validation cohort, respectively (Figure 3G and Figure 4D). The samples in both the training and validation cohorts were subsequently separated into high- and low-risk groups according to the median risk score. Assessments of the survival of the two groups by Kaplan–Meier estimates showed that high-risk patients had a significantly worse overall survival than the low-risk patients in both cohorts (*p* < 0.001) (Figure 4A,E). Moreover, the distribution of risk score, survival time, and survival status of each patient is illustrated in Figure 4B. Among the 10 significant genes in the risk model, *NSUN7*, *RPL15*, *ZCCHC6*, *DCP1B*, and *GPATCH8* were associated with a favorable prognosis, while *DDX23*, *STRAP*, *PRDX1*, *RPL6*, and *DCAF13* were considered to be risk factors (Figure 4C). Subsequently, a nomogram combining these genes was generated, and each patient was assigned a series of scores corresponding to all of the involved genes. Then the 1-, 3-, and 5-year survival probabilities were projected to the final sum of the scores (Figure 4E). 

Since our group previously revealed that a series of pathways (e.g., apoptotic process, PI3K pathway, RNA splicing, rRNA metabolic process, glycolysis) were significantly associated with first-line treatment failure in ES patients, we performed an ssGSEA analysis in 50 hallmark gene sets to determine the relationship between pathway-enrichment and risk scores (Table S5). As shown in Figure 4G, 23 hallmark gene sets were significantly enriched between high- and low-risk patients, in which only protein secretion and bile-acid metabolism was positively associated with better prognosis. A total of ten overrepresenting gene sets are illustrated in Figure S3A (−log_10_(FDR) > 3). Notably, positive correlations between the risk and ssGSEA scores in high-risk patients were shown in 16 gene sets (*p* < 0.05) (Figure 4H, Table S6), where the reactive oxygen species pathway, increased UV response, and glycolysis represent the top three highest correlation coefficients (*p* < 0.001; r = 0.67, 0.61, and 0.60, respectively) (Figure S3B–D). 

### 3.6. Validation of the Prognostic Value and Expression of the RBPs Involved in RPRM

**Figure 5:**
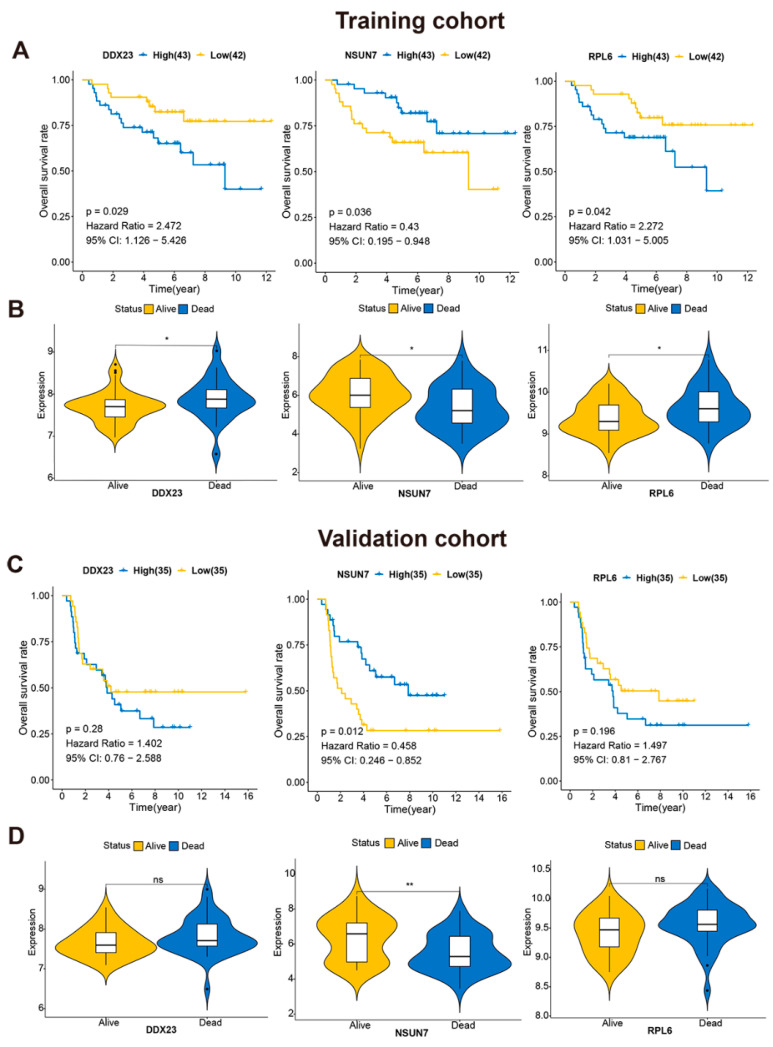
Validation of the prognostic and expression value of the RBPs involved in RPRM in training cohort (**A**). Kaplan–Meier curves show the overall survival in training group with high-risk and low-risk subgroups by *DDX23*, *NSUN7*, and *RPL6*, based on the median value of these genes, respectively. (*p* < 0.05). (**B**). The expression levels of three significant genes in the training group by survival status. The Wilcoxon rank sum test was used to compare the differences between groups. * *p* < 0.05. (**C**). Kaplan–Meier curves show overall survival in validation group with high-risk and low-risk subgroups by *DDX23*, *NSUN7*, and *RPL6* based on the median value of these genes, respectively. (**D**). The expression levels of *DDX23*, *RPL6*, *NSUN7* in validation group by survival status. The Wilcoxon rank sum test was used to compare the differences between groups. ** *p* < 0.01, ns: no significant.

To further assess the independent prognostic value of each key RBPs in ES, the Kaplan–Meier estimates were used to evaluate the relationship between these key RBPs and OS. As a result, six gene expressions (*DCP18*, *DDX23*, *GRATCH8*, *NSUN7*, *RPL6*, and *ZCCHC6*) were identified as being significantly associated with OS in the training cohort: high (≥median) and low (<median). A total of four of these were beneficial to prognosis, and two were associated with poor outcomes (*p* < 0.05) (Figure 5A, Figure S5). The Kaplan–Meier plots of the other remaining four genes are shown in Figure S4 (*p* > 0.05). Additionally, we found that the expression level of *DDX23*, *RPL6*, and *NSUN7* were significantly correlated with survival status (Wilcoxon rank-sum test, *p* < 0.05, Figure 5B), but only *NSUN7* could be verified (log-rank test, Wilcoxon rank-sum test, *p* < 0.05, Figure 5C,D).

**Figure 6:**
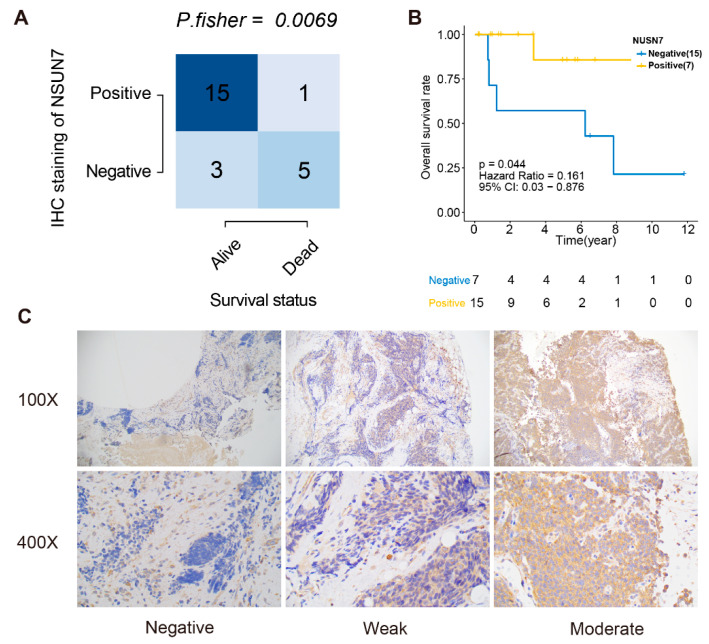
Examples of histology imaging and follow-up in patients with ES. (**A**). Comparison of the survival status and the *NSUN7* IHC scores in 24 ES patients (Fisher’s exact test). (**B**). Kaplan–Meier curve shows the overall survival of *NSUN7* positive and negative in the subgroup of IHC staining. (**C**). Comparison between *NSUN7* immunoreactivity in negative, weak, and moderate scoring at 100× and 400× magnification.

The immunoreactivity of *NSUN7* was scored as negative (*n* = 8), weak (*n* = 6) or moderate (*n* = 10) in 24 patients (Table S7). The proportion of negative cases was higher in pre-treated tissue (7/15) than in therapy-naïve (1/9) biopsies, which may indicate that *NSUN7*-negative cells are more resistant to treatment. In the therapy-naive biopsies, 5/9 cases were classified as good responders according to the treatment protocol (<10% viable tumor cells after treatment), and in the post-therapy specimens, only 2/11 were good responders. We found a significant association between *NSUN7* immunoreactivity and overall survival (Fisher’s exact test, *p* = 0.0069, Figure 6A) and that *NSUN7* negative cases had a shorter overall survival compared to positive ones (log-rank test, *p* = 0.044, Figure 6B). From the preoperative biopsies, only a single patient died (scored as negative for *NSUN7*). The prognostic association across three comparisons (negative vs. weak vs. moderate) are shown in Figure S6 and Figure 6C (chi-square test, *p* = 0.0084; log-rank test, *p* = 0.101).

These results strongly suggest that *NSUN7* may be an important biomarker for ES therapy resistance and that the absence of immunoreactivity may be a prognostic marker.

## 4. Discussion

Our group had previously reported an association between RBP expression and treatment resistance in Ewing sarcoma [22]. Here, we sought to evaluate the putative relationship between RBPs and patient outcome in four previously published transcriptome datasets. We identified three RBP-related transcriptional subtypes that were significantly associated with overall patient survival. We constructed and validated a 10-gene prognostic risk model that showed good clinical applicability as a prognostic marker for ES patients. Finally, one individual transcript in the model (NSUN7) was validated on a protein level in a separate cohort.

It is well known that RBPs are critical for various biological processes. Perhaps not surprisingly, we identified 171 prognostic-associated RBPs that were mostly enriched in RNA (mRNA, ncRNA, rRNA) metabolism, splicing, and translation, and these were similar to our previous findings from the RNA-sequencing of a single Ewing sarcoma cohort. Differential expression between the three RBP-related subtypes also identified 18 transcription factors that significantly co-varied with the 171 RBPs, potentially identifying the regulatory network that define the three RBP subtypes. While many of the identified transcription factors were described as central to cancer development (including SMARCB1, MAZ, EZH1 and H2AFX), none had an established role in ES, likely due to the limited number of functional studies of this tumor type. 

Current chemotherapy protocols can cure a significant proportion of ES patients, but progression under chemotherapy is likely to drive resistance mechanisms. It may therefore be valuable to identify patients with a high likelihood of limited treatment response to offer alternative therapies in clinical trials. Since our 10-gene prognostic model was based on patients with current treatment regimes, our model could be used to identify patients who are at a high risk of death and who derive little benefit from current chemotherapy protocols. 

When considering each gene in the model, only six were significantly associated with patient outcomes in the training cohort. The expression levels of three (*DDX23*, *RPL6*, and *NSUN7*) were related to survival status, but only *NSUN7* was validated as an individual marker, which may be related to the semi-quantitative nature of immunohistochemistry protocols. While this may sound disheartening, it should be interpreted as meaning that multiple gene analyses are required to create a functional prognostic panel for ES. For comparison, validated and implemented prognostic panels for breast cancer contain ~50 genes [42]. 

Consistent with our previous findings, we also found that cancer-related pathways were mostly activated in the ES samples with high-risk scores. Of these, high expression of hypoxia-inducible factor 1 α (HIF1α) emerged as an novel hallmark pathway, which was described to be correlated with a hypoxic tumor microenvironment in ES [43]. These findings are in line with the well-known prognostic value of serum lactate dehydrogenase (LDH) in ES patients [44].

## 5. Conclusions

In summary, this study identified three distinct RBPs signatures in ES. Through DERBP stratification, an RPRM risk model was constructed and validated to be a potential prognostic factor for ES patients. Major limitations of this study should be noted, including a relatively small sample size (155 patients) and the lack of detailed clinical characteristics for many of the cases. However, our results provide a strong rationale for the continued investigation of RBPs since they seem central to chemotherapy resistance and ES patient outcomes.

## Figures and Tables

**Table 1:** GEO datasets and patient characteristics of ES.

GEO ID	No. of ES Cases Included	Platform	Age		Sex		Outcomes	
			<18	≥18	Male	Female	Dead	Alive
GSE63155	46	HuEx1.0 (GPL5175)	41	5	27	19	14	32
GSE63166	39	HuEx1.0 (GPL5175)	31	8	19	20	11	28
GSE17679	32	U133Plus2.0 (GPL570)	16	16	22	10	20	12
GSE34620	38	U133Plus2.0 (GPL570)	27	11	20	18	21	17

## Data Availability

The data presented in this study are available on request from the corresponding author.

## Supplementary Materials

The following are available online at https://www.mdpi.com/article/10.3390/cancers13153736/s1, Figure S1. GEO data processing and normalization (A). A Venn diagram illustrates the overlap of the genes across the four involved GEO datasets of ES. (B,C). The PCA plots show the overall GEO data profiles of GSE17679, GSE34620, GSE61355, and GSE61366 before and after removing batch effect, respectively. (D,E). The box plots show the expression profiles of GSE17679, GSE34620, GSE61355, and GSE61366 before and after removing batch effect, respectively. Figure S2. Consensus matrices of identified clusters (k = 2, 4–6); Figure S3. (A). The distribution of significant gene sets enriched in high-risk patients at cutoff with −log10 (FDR) > 3. (B–D). Violin plots show the correlation between risk score and enrichment scores of reactive oxygen species pathway, UV response up, and glycolysis, respectively. Figure S4. Kaplan-Meier curves show the overall survival in training group with high-risk and low-risk subgroups by DCF13, PRDX1, RPL15, and STRAP, based on the median value of these gene expression levels, respectively. (*p* < 0.05). Figure S5. (A). Kaplan-Meier curves show the overall survival in training group with high-risk and low-risk subgroups by DCP1B, GPATCH8, and ZCCHC6, based on the median value of these gene expression levels, respectively. (*p* < 0.05). (B). The expression levels of DCP1B, GPATCH8, and ZCCHC6 in training group by survival status. The Wilcoxon rank sum test was used to compare the differences between groups. * *p* < 0.05. Figure S6. (A). Comparison of the survival status and the NSUN7 IHC scores (negative vs. weak vs. moderate) in 24 Ewing sarcoma patients (Fisher’s exact test). (B). Kaplan-Meier curve show the overall survival of NSUN7 (negative vs. weak vs. moderate) in the subgroup of IHC staining. Table S1. The top 15 GO biological processes of DERBPs in ES. Table S2. The top 15 REACTOME pathways of DERBPs in ES. Table S3. The top 15 hub genes with the highest degree of connectivity in the PPI network. Table S4. Correlations between RBPs and TFs. Table S5. ssGSEA scores of 50 Hallmarks and risk score in training cohort. Table S6. The correlations between risk score and 23 significant hallmarks. Table S7. IHC scoring and corresponding clinical characteristics in 24 Ewing sarcoma patients’ cohort.
