# Supplementary material for: Identification of an RNA-Binding-Protein-Based Prognostic Model for Ewing Sarcoma
Source: Cancers (Basel). 2021 Jul 25;13(15):3736. doi: 10.3390/cancers13153736 (PMC8345188; doi:10.3390/cancers13153736)
Supplement: Supplementary file 1 [file cancers-13-03736-s001.zip › Supplementary files/Table S1.pdf]

GO term (biological process)	No. of genes	Term *P*.Value
RNA processing	126	3.37E-103
mRNA processing	79	6.02E-65
mRNA metabolic process	92	3.74E-63
RNA splicing	69	1.48E-56
ncRNA metabolic process	57	7.09E-40
translation	61	2.15E-36
ncRNA processing	46	7.80E-32
ribonucleoprotein complex assembly	35	1.41E-30
posttranscriptional regulation of gene expression	55	4.74E-30
regulation of translation	38	2.06E-22
RNA 3'-end processing	24	1.42E-19
RNA catabolic process	35	1.88E-19
rRNA processing	26	2.29E-18
mitochondrial translation	21	1.46E-17
regulation of RNA splicing	22	6.55E-17
