# Supplementary material for: Identification of an RNA-Binding-Protein-Based Prognostic Model for Ewing Sarcoma
Source: Cancers (Basel). 2021 Jul 25;13(15):3736. doi: 10.3390/cancers13153736 (PMC8345188; doi:10.3390/cancers13153736)
Supplement: Supplementary file 1 [file cancers-13-03736-s001.zip › Supplementary files/Table S2.pdf]

Reactome pathways	No. of genes	Term *P*.Value
Metabolism of RNA	74	7.20E-54
Processing of Capped Intron-Containing Pre-mRNA	40	2.53E-34
mRNA Splicing	36	4.40E-33
mRNA Splicing - Major Pathway	33	1.27E-29
Translation	31	1.08E-20
mRNA Splicing - Minor Pathway	16	9.56E-19
Mitochondrial translation	18	5.41E-17
Mitochondrial translation termination	16	7.59E-15
Mitochondrial translation elongation	16	7.59E-15
Mitochondrial translation initiation	16	7.59E-15
RNA Polymerase II Transcription Termination	13	1.38E-12
Deadenylation-dependent mRNA decay	12	2.36E-12
RNA polymerase II transcribes snRNA genes	12	9.65E-11
mRNA 3'-end processing	11	1.02E-10
rRNA modification in the nucleus and cytosol	10	4.67E-09
