# Supplementary material for: Identification of an RNA-Binding-Protein-Based Prognostic Model for Ewing Sarcoma
Source: Cancers (Basel). 2021 Jul 25;13(15):3736. doi: 10.3390/cancers13153736 (PMC8345188; doi:10.3390/cancers13153736)
Supplement: Supplementary file 1 [file cancers-13-03736-s001.zip › Supplementary files/Table S3.pdf]

RBP gene symbol	Gene description	No. of edges (degree)
EIF4A3	Eukaryotic initiation factor 4A-III	54
POLR2F	DNA-directed RNA polymerases I, II, and III subunit RPABC2	49
SRSF1	Serine/arginine-rich splicing factor 1	48
POLR2E	DNA-directed RNA polymerases I, II, and III subunit RPABC1	47
POLR2L	DNA-directed RNA polymerases I, II, and III subunit RPABC5	47
SNRPB	Small nuclear ribonucleoprotein-associated proteins B and B	47
POLR2I	DNA-directed RNA polymerase II subunit RPB9	46
PCF11	Pre-mRNA cleavage complex 2 protein Pcf11	45
POLR2G	DNA-directed RNA polymerase II subunit RPB7	45
SNRPF	Small nuclear ribonucleoprotein F	45
PRPF19	Pre-mRNA-processing factor 19	44
SF3B1	Splicing factor 3B subunit 1	44
RNPS1	RNA-binding protein with serine-rich domain 1	41
CSTF3	Cleavage stimulation factor subunit 3	40
SRSF11	Serine/arginine-rich splicing factor 11	40
