# Supplementary figures and images for: Identification of an RNA-Binding-Protein-Based Prognostic Model for Ewing Sarcoma
Source: Cancers (Basel). 2021 Jul 25;13(15):3736. doi: 10.3390/cancers13153736 (PMC8345188; doi:10.3390/cancers13153736)

**A**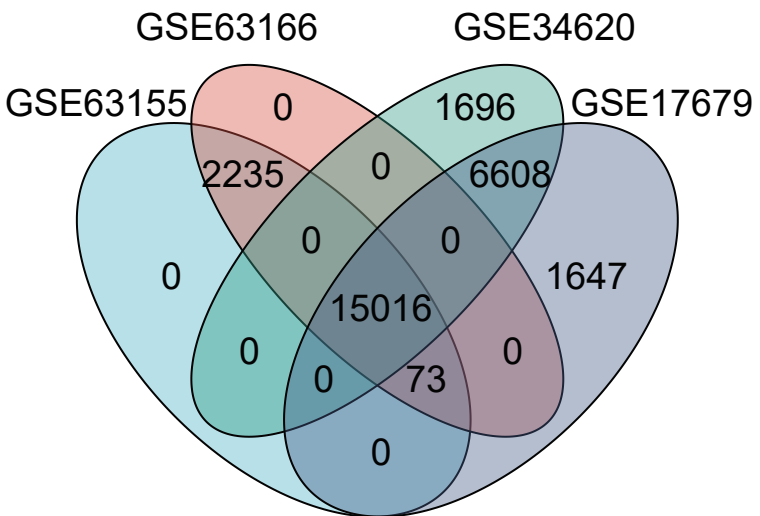**B**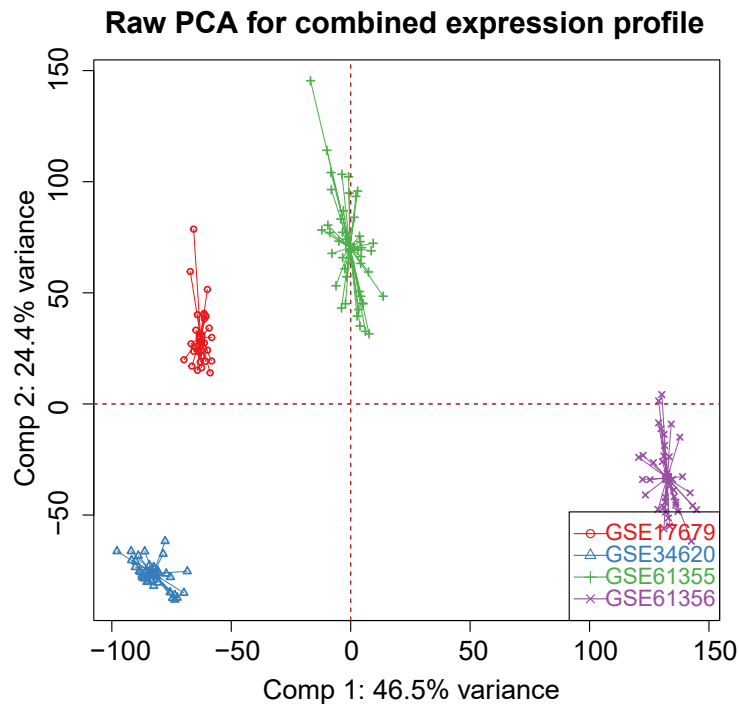**C**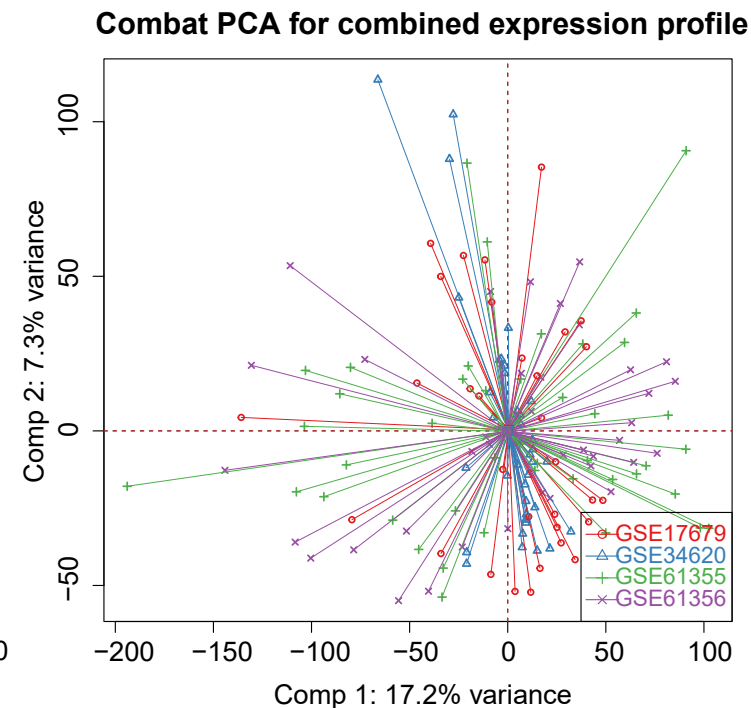**D**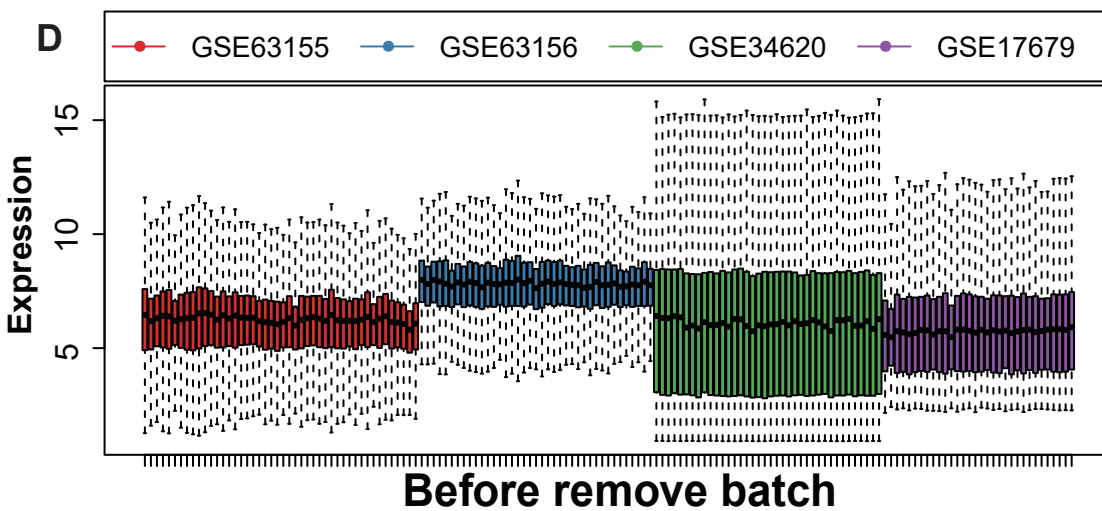**E**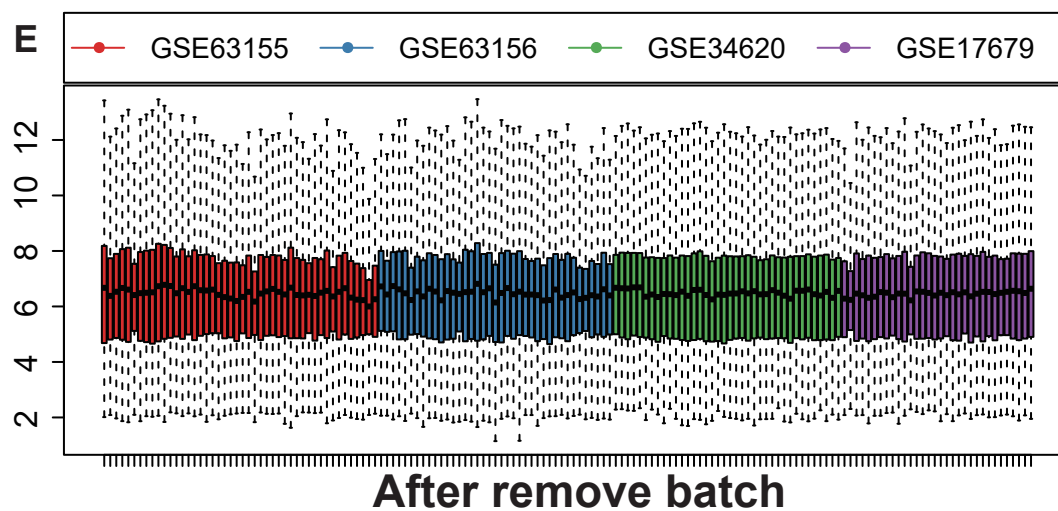

Supplement: Supplementary file 1 [file cancers-13-03736-s001.zip › Supplementary files/Figure S1.pdf]

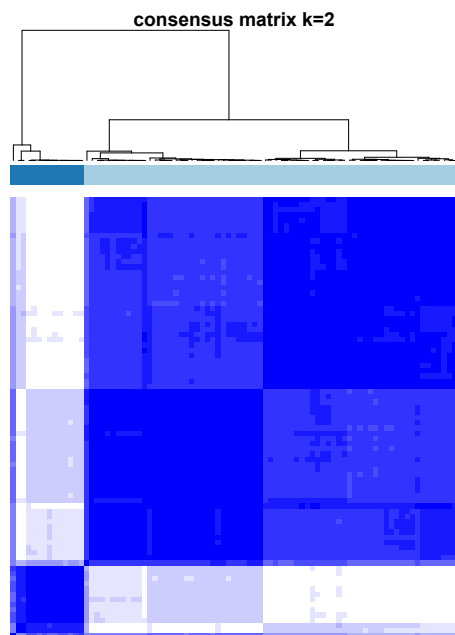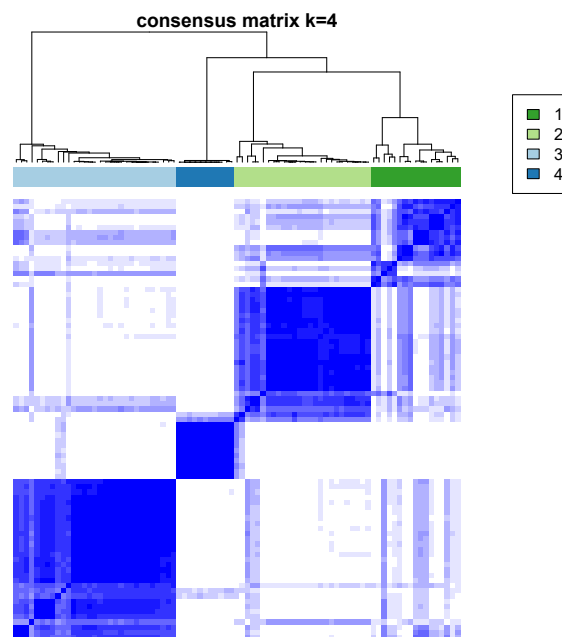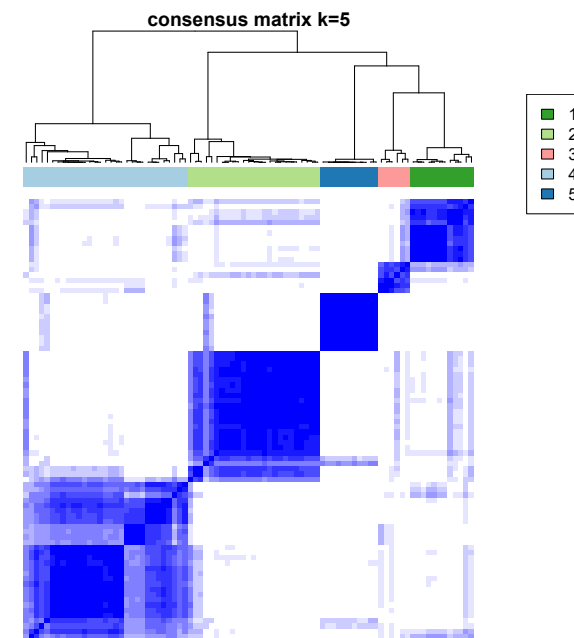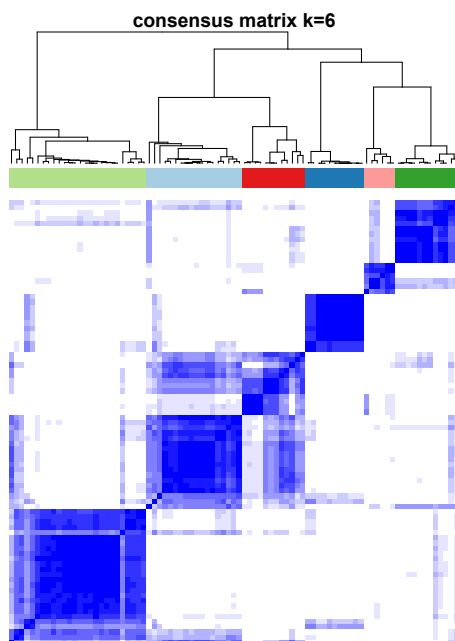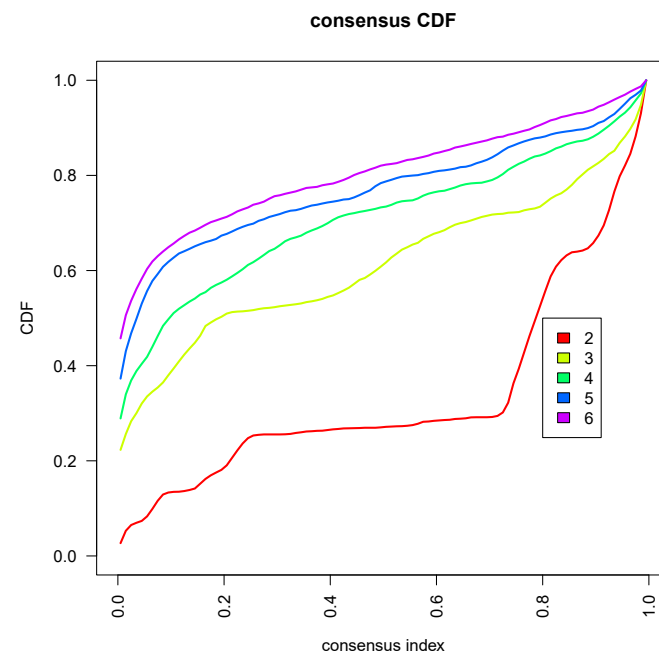

Supplement: Supplementary file 1 [file cancers-13-03736-s001.zip › Supplementary files/Figure S2.pdf]

**A**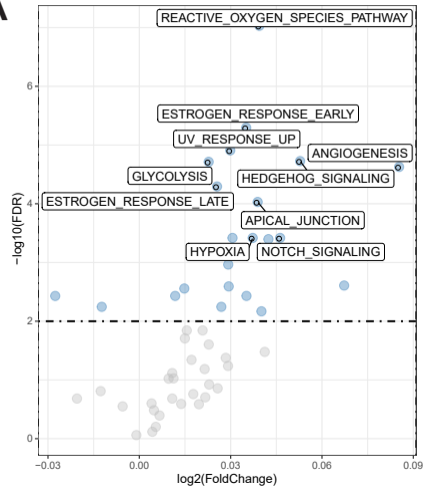**B**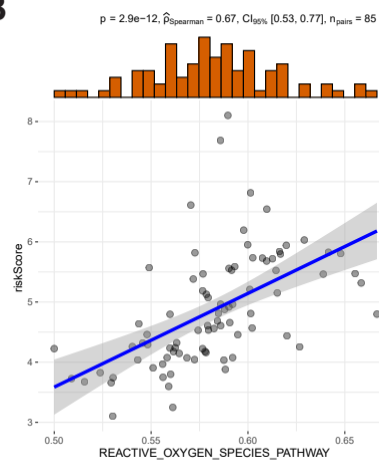**C**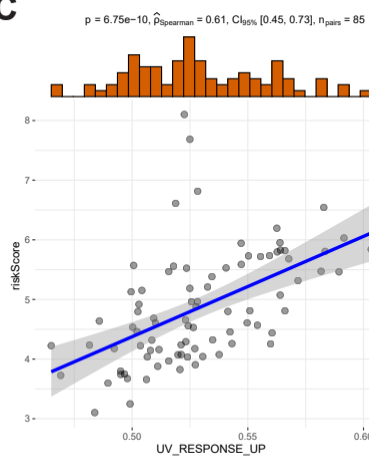**D**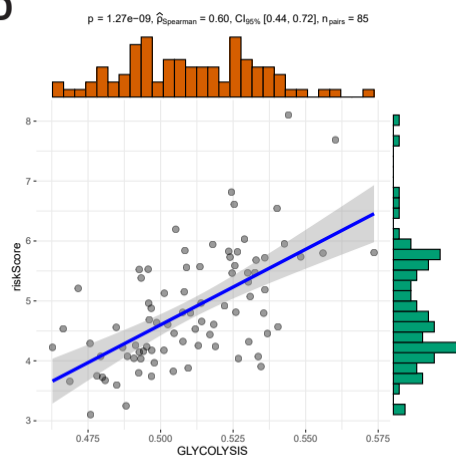

Supplement: Supplementary file 1 [file cancers-13-03736-s001.zip › Supplementary files/Figure S3.pdf]

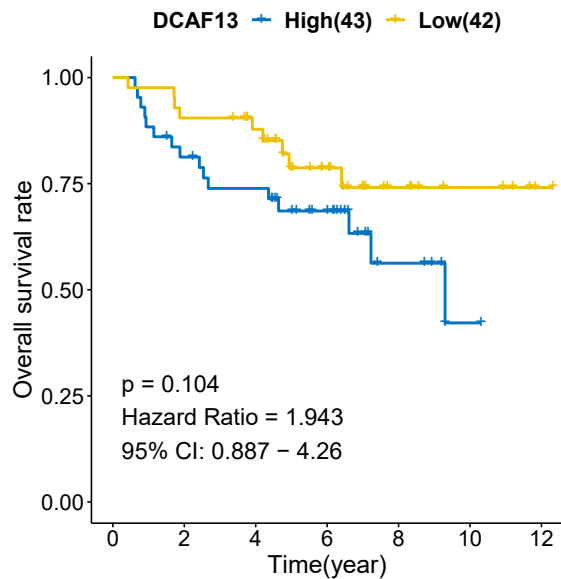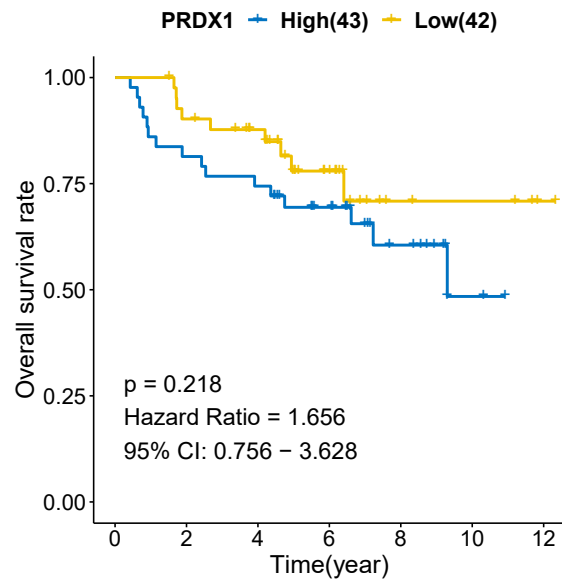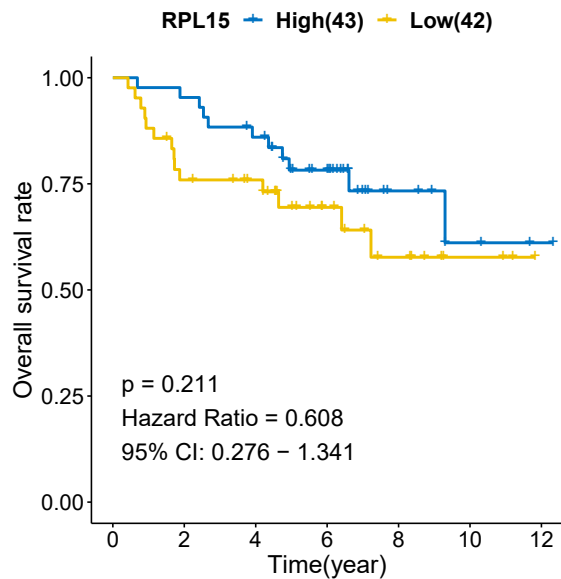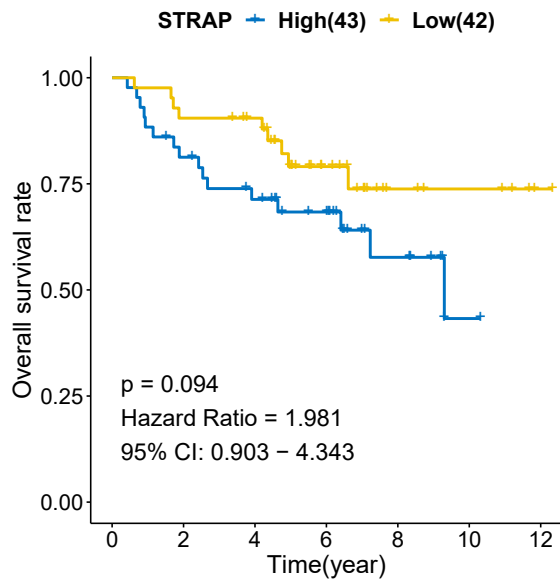

Supplement: Supplementary file 1 [file cancers-13-03736-s001.zip › Supplementary files/Figure S4.pdf]

A

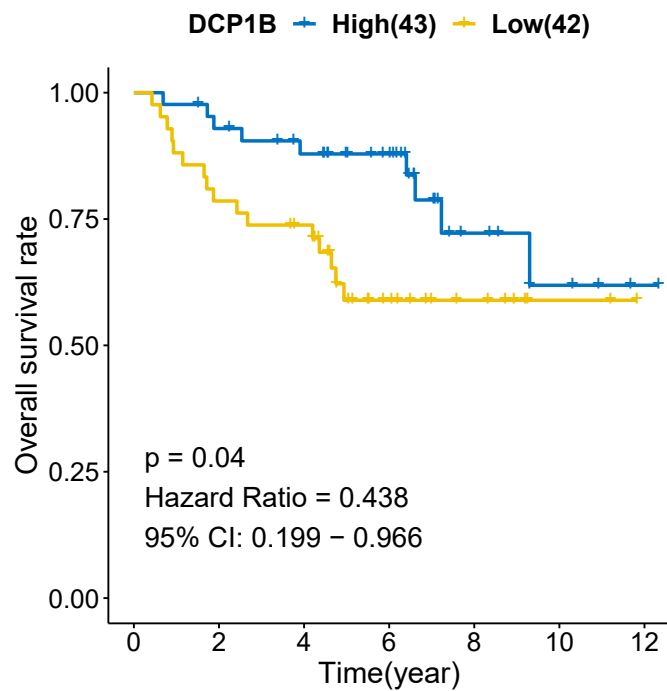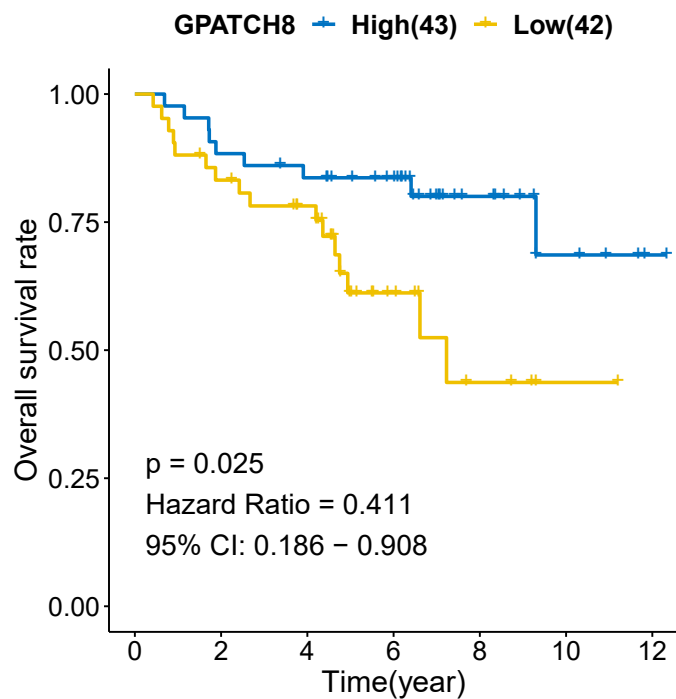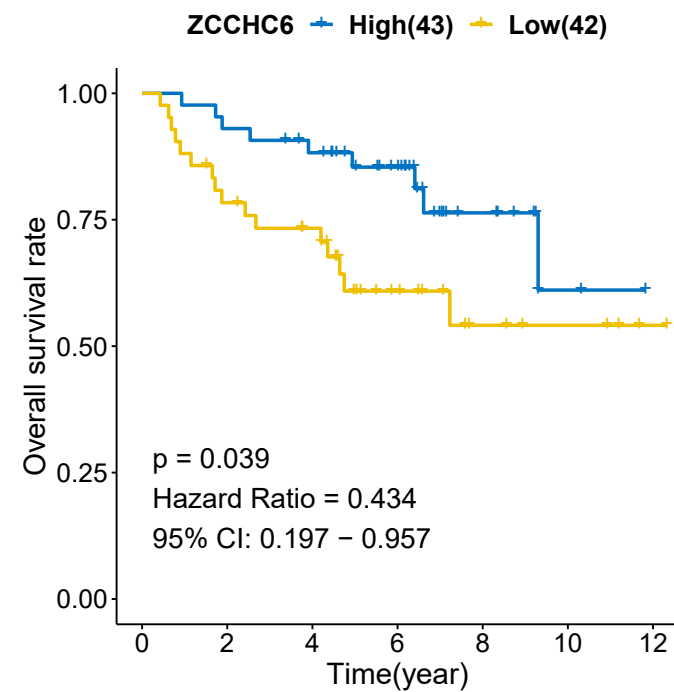

B

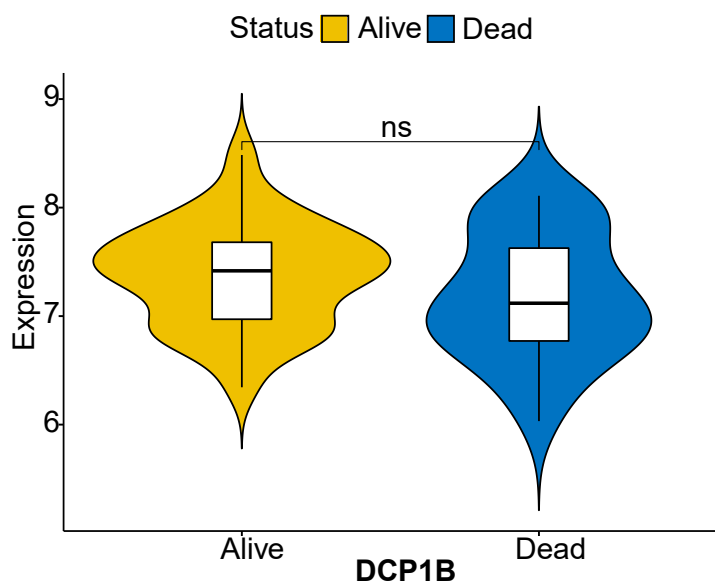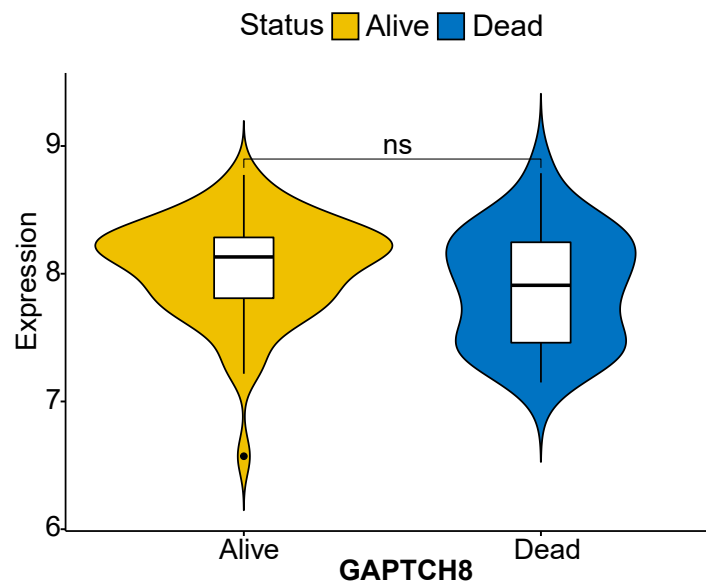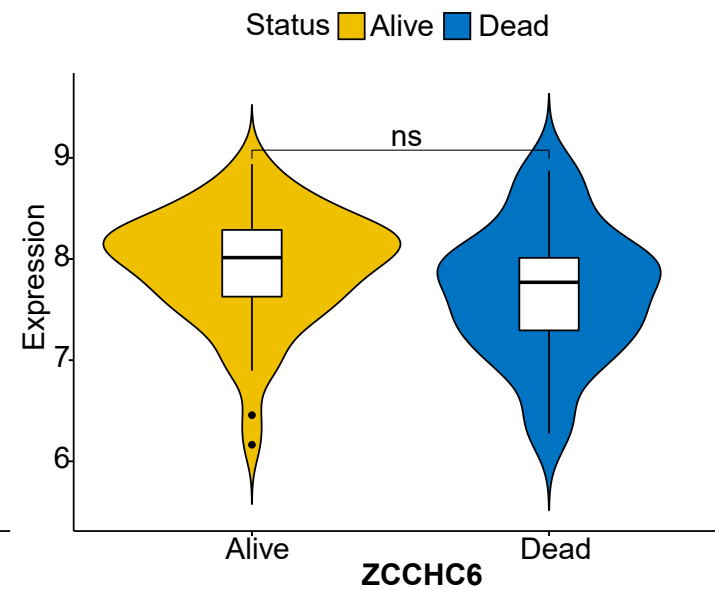

Supplement: Supplementary file 1 [file cancers-13-03736-s001.zip › Supplementary files/Figure S5.pdf]

A

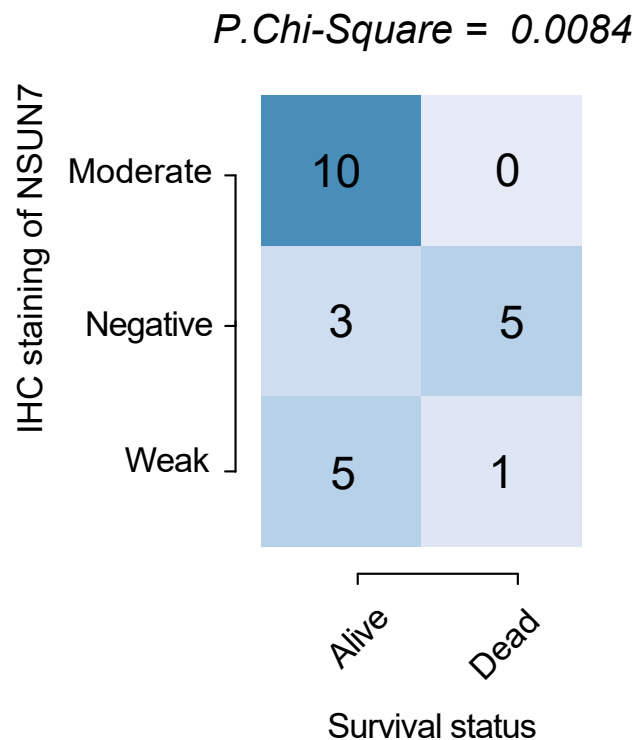

B

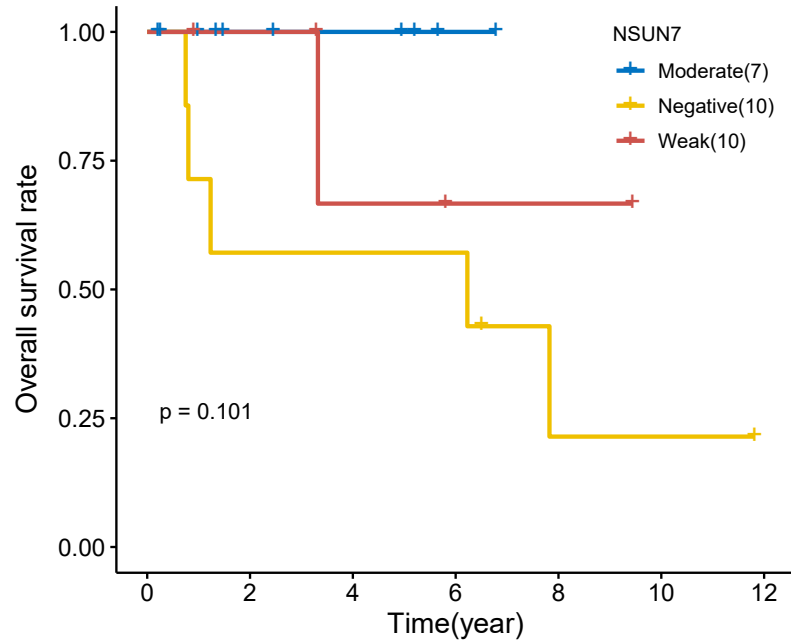

|          |    |   |   |   |   |   |   |
|----------|----|---|---|---|---|---|---|
| Moderate | 10 | 5 | 4 | 1 | 0 | 0 | 0 |
| Negative | 7  | 4 | 4 | 4 | 1 | 1 | 0 |
| Weak     | 5  | 4 | 2 | 1 | 1 | 0 | 0 |

Supplement: Supplementary file 1 [file cancers-13-03736-s001.zip › Supplementary files/Figure S6.pdf]
